# Improved identification of conserved cassette exons using Bayesian networks

**DOI:** 10.1186/1471-2105-9-477

**Published:** 2008-11-12

**Authors:** Rileen Sinha, Michael Hiller, Rainer Pudimat, Ulrike Gausmann, Matthias Platzer, Rolf Backofen

**Affiliations:** 1 Genome Analysis, Leibniz Institute for Age Research – Fritz Lipmann Institute, Beutenbergstr. 11, 07745 Jena, Germany; 2 Bioinformatics group, Albert-Ludwigs-University Freiburg, Georges-Koehler-Allee 106, 79110 Freiburg, Germany; 3 Department of Developmental Biology, Stanford University, Stanford, CA 94305, USA

## Abstract

**Background:**

Alternative splicing is a major contributor to the diversity of eukaryotic transcriptomes and proteomes. Currently, large scale detection of alternative splicing using expressed sequence tags (ESTs) or microarrays does not capture all alternative splicing events. Moreover, for many species genomic data is being produced at a far greater rate than corresponding transcript data, hence *in silico *methods of predicting alternative splicing have to be improved.

**Results:**

Here, we show that the use of Bayesian networks (BNs) allows accurate prediction of evolutionary conserved exon skipping events. At a stringent false positive rate of 0.5%, our BN achieves an improved true positive rate of 61%, compared to a previously reported 50% on the same dataset using support vector machines (SVMs). Incorporating several novel discriminative features such as intronic splicing regulatory elements leads to the improvement. Features related to mRNA secondary structure increase the prediction performance, corroborating previous findings that secondary structures are important for exon recognition. Random labelling tests rule out overfitting. Cross-validation on another dataset confirms the increased performance. When using the same dataset and the same set of features, the BN matches the performance of an SVM in earlier literature. Remarkably, we could show that about half of the exons which are labelled constitutive but receive a high probability of being alternative by the BN, are in fact alternative exons according to the latest EST data. Finally, we predict exon skipping without using conservation-based features, and achieve a true positive rate of 29% at a false positive rate of 0.5%.

**Conclusion:**

BNs can be used to achieve accurate identification of alternative exons and provide clues about possible dependencies between relevant features. The near-identical performance of the BN and SVM when using the same features shows that good classification depends more on features than on the choice of classifier. Conservation based features continue to be the most informative, and hence distinguishing alternative exons from constitutive ones without using conservation based features remains a challenging problem.

## Background

Eukaryotic primary mRNAs consist of exons and introns. The mature transcript as the substrate for translation is produced by removing introns in a process called splicing. Splicing can be either constitutive, always producing the same mRNA, or alternative, by skipping of variable parts of the primary transcript.

Alternative splicing is a mechanism for producing transcript and protein diversity [1]. It is particularly widespread in higher eukaryotes, especially mammals. Various studies have estimated that up to 74% of all human genes are alternatively spliced. Large scale detection of alternative splicing is usually done using expressed sequence tags (ESTs) [2] or microarrays (reviewed in [3] and [4]). Since alternative splicing can be highly specific for tissues or developmental stages, these methods can only detect splice events that occur in the underlying probe samples with sufficient frequencies and/or are limited to by the microarray design. Furthermore, nowadays Whole Genome Shotgun (WGS) sequencing projects are churning out genomic data at a higher rate than corresponding transcriptome data – the number of ESTs in GenBank Release 161 had increased by 19% in one year, compared to a gain of 39% in the number of contigs in the WGS GenBank division [5]. Thus it can be expected that in the foreseeable future, we shall have several genomes without the level of corresponding extensive transcript coverage required to reveal the extent of alternative splicing, and hence transcriptomic and proteomic variability. Accordingly, there is a need for *in silico *methods of detecting alternative splicing. Moreover, such methods can provide further insights into the mechanisms of alternative splicing.

Exon skipping, whereby a given exon in its entirety is either included in, or excluded from the mature transcript, is the most prevalent form of alternative splicing in humans [6]. It has been shown that sequence-based features, derived from the exon and its flanking introns, can be used to predict skipping of exons that are conserved between human and mouse and alternatively spliced in both species; denoted conserved exon skipping events [7]. Previous studies have used such features with state-of-the-art classifiers such as support vector machines (SVMs) [8,9] and regularized least-squares classifier [10], and achieved success in predicting exon skipping. Other approaches use protein domain information [11] and evolutionary conservation [12-14] to detect alternative splice events.

Here, we use Bayesian networks (BNs), a state-of-the-art machine learning method, to predict conserved exon skipping events. BNs are an increasingly popular machine learning approach to data modeling and classification [15,16]. The ability of BNs to cope with features of various value ranges and to learn dependencies between features makes them especially versatile and suited to a large variety of applications. BNs allow multiple dependencies between variables, impose no fixed ordering of variables, allow integration of arbitrary features, and the network structure can be automatically learned. This makes BNs a flexible choice for biological sequence data analysis [17-21]. We introduce several novel features that distinguish alternative exons from constitutive exons, including features based on the single-strandedness of exonic splicing enhancers and silencers (ESEs and ESSs), and features involving intronic splicing regulatory elements (ISREs). By validating our classifiers on various datasets, we identify features which are discriminative irrespective of dataset-specific biases, and provide independent measures of the predictive power of the BNs.

Even though conservation based features have proved to be among the most discriminative features for predicting exon skipping, it is desirable to be able to predict alternative splicing using only information from a single genome. We show that our approach can still predict exon skipping without using conservation-based features.

## Methods

### Datasets and genome browser

We used the dataset of [8], henceforth called dataset D1, consisting of 243 alternative and 1,753 constitutive exons, kindly provided by Gideon Dror. In this dataset, constitutive exons are supported by at least four ESTs each in human and mouse with no EST evidence for exon skipping, whereas alternative exons are skipped in both species. The second dataset is the ACESCAN training set [10], henceforth called dataset D2, which comprises 5,069 constitutive and 241 alternative exons. For validation purposes we use the genome builds hg18 for human and mm9 for mouse of the UCSC Genome Browser [22] were used.

### Features for machine learning

In total, we used 365 features in this study (Table 1). Thereof, 228 were previously used by [8]: (1) exon length, (2) symmetry, that is, divisibility of exon length by 3; (3) percent identity of the alignment between the exon and its mouse ortholog; (4–7) length of and percent identity of the best local alignment between the up- and downstream 100 nt intronic flanks and their mouse orthologs, which are in total four features; (8–199) trimer counts for the exon and the 100 nt flanking intronic regions, which are a total of 64 × 3 = 192 features; (200) intensity of the poly-pyrimidine tract (PPT) as the number of pyrimidines in the window -19 to -4 from the 3'ss; and (201–228) nucleotides at the 5'ss positions -3 to -1 and +3 to +6, which are a total of 7 × 4 = 28 features. The features (1–7), used for the first time in [7], were kindly provided by Gideon Dror, along with the dataset D1.

**Table 1:** Features for machine learning used in this study

**Feature subset**	**Number of features**	**Motivation**	**First use**
Exon: length, symmetry, and identity with mouse ortholog	3	Alternative exons tend to be shorter, frame-preserving, and more conserved compared to constitutive exons	[7]
Conservation of intronic flanks: length/identity of the best local and identity of the global alignment	2 × 3	Alternative exons tend to have higher conservation in their intronic flanks	[7,10]
Conservation in a 12 nucleotide region spanning the 3' and 5'ss	2	As alternative exons and their intronic flanks are more conserved, this may in particular concern the exon/intron boundaries	This work
PPT intensity	1	Alternative exons tend to have weaker PPTs	[8]
Nucleotides at seven positions flanking the 5'ss	4 × 7	Alternative exons tend to have specific nucleotide preferences near the 5'ss	[8]
Frequency of di- and trimers in the exon and flanking introns	3 × 16 3 × 64	Motifs which are part of splice regulatory motifs might differ in their abundance in alternative and constitutive exons	[8] (trimers), this work (dimers)
Splice site strength of 3'and 5'ss	2	Alternative exons tend to have weak splice sites	[10]
Length of flanking introns	2	Alternative exons tend to be flanked by long introns	[10]
GC content of exon and intronic flanks	3	GC-poor regions tend to promote alternative splicing	This work
Features based on NI scores	24	Alternative exons tend to have fewer ESEs and more ESSs	This work
Features based on PU values	15	Single-stranded motifs are likelier to bind to regulators	This work
PTB-binding sites	6	PTB is a regulator alternative splicing	This work
Features based on ISREs	8	Alternative exons tend to have more ISREs in their intronic flanks	This work
Density of various motifs	22	Several motifs are known to be associated with alternative splicing	This work
Combination features	7	Combining features can capture more information	This work

Note that the total number of features used is 365 whereas the sum of the entries here is 378, because some features have been counted in more than one category (for example, in PU value and NI score related features).

We also used six features from [10]: the percent identity of the global alignments between up- and downstream 100 nt intronic flanks and their mouse orthologs, lengths of the upstream and downstream flanking introns, and the strength of the 3' and 5' splice sites (3'ss and 5'ss) computed by MAXENTSCAN [23]. We used the programs "needle" and "water" from the EMBOSS software suite [24] for aligning the exons and the intronic flanks with their mouse orthologs and computing the conservation based features.

Among the new features we added were dinucleotide counts for the exon and the 100 nt intronic flanks, a total of 16 × 3 = 48 features. As it has been shown that exon skipping is more prevalent in regions of low GC content [25], we used the GC content of the exon and the intronic flanks as three additional features.

To use features based on ESEs and ESSs, we applied neighbourhood inference (NI) scores [26]. Briefly, each hexamer has an NI score between -1 and 1, with negative scores indicating a tendency towards acting as an ESS, and a positive score, a tendency to act like an ESE. Hexamers with a score of 1 or -1 are considered "trusted" ESEs and ESSs, respectively, and those with a score of greater than 0.8 or smaller than -0.8 are considered to have "strong" ESE or ESS activity. We used the density of NI scores, defined as the number of hexamers with NI scores 1, ≥ 0.8, > 0, < 0, ≤ -0.8, -1, normalized by the number of hexamers in the exon (6 features). Additionally, the distribution of ESEs and ESSs may have a bearing on splicing as well. Therefore, we used the variance of NI scores for "trusted" and "strong" ESEs and ESSs (2 features). Since the density of ESEs and ESSs near splice junctions has been suggested to be important in determining splicing outcome [4,27-29], we also measured the densities in the first and last 50 nucleotides of the exon (for exons shorter than 50 nt, the entire exon was used; 2 features).

We also designed features using very recently published datasets of conserved ISREs enriched in the upstream and downstream intron flanks of all exons, as well ISREs enriched in upstream and downstream introns flanking alternative exons [30]. We used the density of ISREs from these four lists in both upstream and downstream 100 nt flanking intronic regions, giving us eight novel features.

Secondary structure can influence alternative splicing [31]. The single-strandedness of ESE, ESS or ISRE motifs was characterized using PU (Probability of being Unpaired) values [32], which represent the probability that all the bases in the given motif are unpaired. Since local RNA folding is influenced by the length of the sequence context [33], we minimized potential biases by using 11 to 30 nt symmetrical context lengths up- and downstream of a given hexamer, and computing the average of the 20 PU values thus obtained [34]. We pre-computed PU values in this manner for all the hexamers in the exons, and combined the NI scores with PU values. Various thresholds were used for absolute NI score value (1, = 0.8, > 0) and a PU value of 0.6. Two kinds of combinations were used: (i) a "Boolean" combination, that is, counting the number of hexamers with NI and PU values both above the thresholds; and (ii) the product of NI and PU values (4 features). Similarly, we used PU values in conjunction with ISRE information to characterize the single-strandedness of intronic splicing regulatory elements (4 features).

Mutations around the splice junctions can effect splicing. Therefore, we designed a feature to measure how well the immediate neighbourhood of the splice junctions was conserved. We formed two 12-mers consisting of the bases from positions -6 to +6 around the 3'ss and the 5'ss. The number of identical nucleotides between the human and mouse 12-mers result in two new features.

We also used several motifs from a recent study characterizing conserved motifs associated with constitutive and alternative splicing [35]. However, depending on the partition, these features were either not discriminative or weakly so, indicating that they are important only for a small minority of the alternative exons.

To count the number of binding sites for the Polypyrimidine-tract-binding protein (PTB), a well-studied repressive regulator of alternative splicing [36], we counted the simplest known motifs for its binding sites – UCUU and CUCUCU, as well as the sum. The density of PTB binding sites in the 100 nt intron flanks and the exon gives nine features.

Lastly, we used novel features derived from features already known to be discriminative. For example, while it is known that skipped exons tend, on the average, to be shorter than constitutive exons, it has been shown that long exons can be skipped if flanked by very long introns [37]. Furthermore, it is possible that the shorter the exon is with respect to the flanking introns, the harder it is for the spliceosome to reliably recognize it. Consequently, we used the ratio of upstream and downstream intron length to exon length, as well of the intron lengths, as three features. We also used the pairwise products of human-mouse identity of the exon and each 100 nt intron flank as well as of the exon and both flanks, in order to capture information about simultaneous conservation of the exon and the intronic flanks (four features).

### Information gain and information gain ratio

To compare the information content of the features, we used information gain, and information gain ratio, which are established measures of the usefulness of features in the field of machine learning [38]. The formula for information gain is:

IG(Class | Feature) = H(Class) - H(Class | Feature)

where H(Class) is the entropy of the class variable, and H(Class | Feature) is the conditional entropy of the class variable, given the feature. We used the WEKA package [38] for computing information gain and information gain ratio, in order to rank the features according to how informative they were.

### Bayesian networks

We used the algorithms for feature selection, model learning and classification as described in [17], and made available *via *the public webserver BioBayesNet [39].

### Feature subset selection

Given a training set, we selected features in a three step procedure. First, we use an entropy based method developed by [40] to find partitions of the feature ranges which best separate the given classes (in the following called "discretizer"). Features for which the entire feature range is partitioned into at least two intervals, such that the distributions of the two classes differ significantly in these intervals, are called "discriminative" and they are the basis of further analysis. On the other hand, those features for which no such intervals are found are essentially non-informative, or "non-discriminative" features for our purposes.

Once the discretization algorithm has chosen the set of discriminative features, an optimal (in the local sense) subset can be selected using the sequential floating feature selection (SFFS) method [41]. Briefly, this algorithm starts with an initially empty feature subset, and at each step, adds the feature which most improves a specific quality measure. After this addition, all previously added features are deleted from the subset, unless doing so worsens the quality measure. This is done in order to avoid getting trapped in local minima. The algorithm stops when neither inserting new features nor deleting existing ones improves the quality measure provided by the subset.

Thirdly, one can enforce inclusion or exclusion of any given feature manually. The manual feature selection consists only of removing a few "weak" features (as measured by low information gain, or negligible information loss when they are omitted for classification purpose) as they are unlikely to generalize well to unseen data; and addition of a few "strong" features (as measured by high information gain), which were selected by the discretizer but not by the SFFS algorithm.

### Learning the Bayesian network

We restrict the structure of the BNs by using the so-called tree-augmented naïve Bayes (TAN) structure [42]. In a naïve Bayes classifier/network, the attributes are assumed to be independent, given the class, that is, the node representing the class variable is a parent of all other nodes, and there are no other edges in the network. A TAN classifier augments the underlying naïve Bayes classifier by allowing at most one additional parent per node, that is, each node is the child of the class attribute node, and of at most one more node. We use TAN classifiers because while learning the best BN structure, given some training data, is in general an NP-hard problem [43], for TAN networks there exist efficient structure-learning algorithms that reduce the problem of determining the optimal tree structure to finding a maximum-weighted spanning tree [44]. Once the structure of the network has been learned, the (conditional) probability distributions over the feature values of each feature (given the class label and optionally the value of the parent feature) are estimated in a straightforward manner from count statistics derived from learning data. Finally, Bayesian inference of marginal probabilities can be approximately calculated by the efficient technique of variable elimination [45].

### Data partition

Given a dataset (D1 or D2), we partitioned the data into three equal parts as carried out in [8]. Then, in turn, we used two-thirds of the data to train the BNs, and the remaining one-third was used for testing. The test set remained untouched while the training set was used for discretization, feature selection, and learning the BN [39]. Finally, the BN which had been learned on the training set was used to classify the samples in the test set. This procedure was repeated twice for the other two one-thirds, and the average of the three runs was taken as the final performance. For comparing 2-fold, 3-fold, 5-fold and 10-fold cross-validation, we used WEKA [38].

## Results and discussion

### Improved prediction of conserved cassette exons by Bayesian networks

As pointed out by [8], good performance at low false positive rates is especially important for the task of distinguishing alternative exons from constitutive exons on a genome-wide scale, since the latter comprise the majority of exons. Furthermore, a low number of false positives is critical in case of experimental verification of predictions. To this end, we measure the true positive rate (TP) at false positive rate (FP) of 0.5%, and call it TP_0.5_. We also compute the receiver operating curve (ROC) and measure the area under the ROC curve (AUC), which is a standard measure of the quality of a classifier [46].

**Figure 1:**
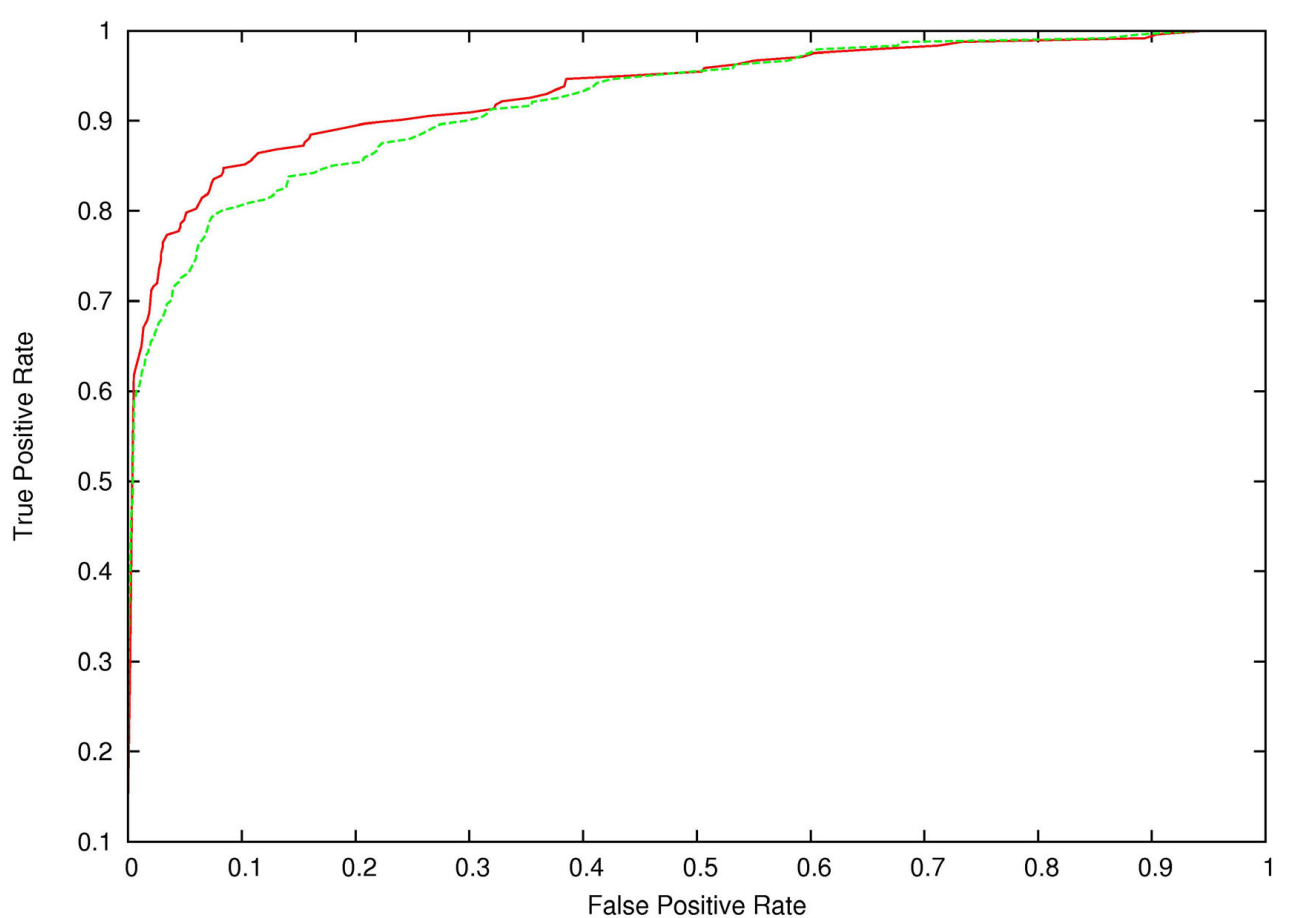
ROC plot showing the average performance of the 3-fold cross-validation on datasets D1 (red line) and D2 (green line).

We used the dataset and the cross-validation scheme described in [8]. This dataset contains 243 alternative and 1,753 constitutive exons and is called D1 in the following. The overall performance obtained, using novel features in addition to those described in the literature (Table 1), was TP_0.5 _= 61%, and AUC = 0.94 (Figure 1), compared to TP_0.5 _= 50%, and AUC = 0.93 reported in [8] using SVMs. This substantial improvement demonstrates that many of the novel features are informative and discriminative for conserved exon skipping events.

### Feature selection

The number of features studied in machine learning tasks is often very high, and many (possibly most) of them might be irrelevant, or redundant [38]. Therefore, it is customary to preprocess the data in order to select a useful subset of features – this is called "feature selection". Feature selection can be carried out in three stages within the BioBayesNet framework [39]. Firstly, a "discretizer" applying the algorithm of Fayyad and Irani [40] discards features for which no suitable discriminative intervals are found. Secondly, the sequential feature subset selection (SFFS) algorithm [41] can be applied to select a subset of the remaining features. Thirdly, one can enforce inclusion or exclusion of any given feature manually. The manual feature selection (on D1 and D2) typically involved the addition and removal of 5 or fewer features each, given a feature subset of 20–30 features obtained using the two automated approaches.

The performance on D1 using only feature selection using the "discretizer" was TP_0.5 _= 39%, and AUC = 0.93. Using the SFFS algorithm for further feature selection resulted in TP_0.5 _= 47%, and AUC = 0.94, whereas the manual inclusion/exclusion of features gave the final performance of TP_0.5 _= 61%, and AUC = 0.94. This illustrates that the overall quality of classification, as measure by the AUC, is quite robust, and we get good performance even when only the "discretizer" is used, but the performance at low false positive rates is quite sensitive to small changes in the feature subset, so the other two methods of feature selection result in significant improvement. We note that manual feature selection is only needed to improve TP_0.5 _– if we consider more global measures of classification performance such the AUC or balanced sensitivity and specificity, the automated feature selection methods suffice. Using only automated feature selection, we routinely achieve AUC values in the 0.93–0.96 range, and balanced sensitivity and specificity in the 87%–91% range.

### Discriminative features

ESEs and ESSs are motifs bound by proteins which either enhance or suppress splicing. It has been shown that alternative and constitutive exons differ in the density of ESEs and ESSs [29]. We used Neighborhood Inference (NI) scores to infer ESE and ESS activity for all hexamers [26]. We used the density of ESEs and ESSs, with various thresholds for the NI scores. The constitutive exons have a slightly higher density of ESEs than do alternative exons (median 0.266 vs. 0.254), as well as ESSs (median 0.0694 vs. 0.0679) This was also confirmed using other ESE/ESS datasets [27,29] and is in agreement with previous studies [26,27,29,30]. Depending on the split, the density of ESEs and ESSs was either not discriminative, or weakly so. Varying the threshold of the NI score did not change this. On the other hand, some of the novel features using NI scores were discriminative on most splits – for instance, the average of all positive NI scores, as well as the average of all negative NI scores. Similarly, the average of all "strong ESEs" (NI score ≥ 0.8) and "strong ESSs" (NI score ≤ -0.8) were discriminative features. However, the density of ESEs and ESSs near the splice sites was not found to be discriminative.

Splicing regulatory elements are found in introns as well [47]. Consequently, we also designed features using very recently published datasets of conserved intronic splicing regulatory elements (ISREs) [30]. Similar to ESE and ESS based features, we also used the density of ISREs in the upstream and downstream intronic flanks. Four sets of ISREs (enriched in the upstream and downstream flank of all exons as well as enriched in the flanks of alternative exons) are given in [30]. We found three of these sets to be discriminative (ISREs from downstream intronic flanks of all exons were not discriminative). For these three discriminative features, alternative exons have a higher density of ISREs than constitutive exons, which agrees well with the finding that the set of ISREs has an overlap with ESSs, and thus many of them may have silencing tendencies [30].

Secondary structure can influence alternative splicing [31]. To the best of our knowledge, existing methods to predict alternative splice events do not use secondary structure related properties. Previously, we found that functionally important splicing motifs are preferentially located in single-stranded mRNA secondary structures [34] and that *ab initio *motif finding benefits from taking the single-strandedness of motif occurrences into account [48]. Thus, we use features based on a measure of the single-strandedness of ESE, ESS or ISRE occurrences, reasoning that single-strandedness of enhancer and silencer occurrences influence the propensity of proteins to bind them. Interestingly, we found that the single-strandedness of ESE motifs is informative. The density of single-stranded ESEs is higher in constitutive than in alternative exons (0.0194 vs. 0.0177, using a PU value of 0.5 for single-strandedness). Moreover, the information gain of this feature was more than that of the ESE density feature (0.0170 vs. 0.0096). As single-stranded motifs are expected binding sites for splicing regulatory proteins, this observation adds to previous evidence that mRNA secondary structures influence alternative splicing [34].

The density of PTB binding sites in the exon and the upstream 100 nt intronic flank were weakly discriminative, indicating that they are important only for a small minority of the alternative exons. The density of PTB binding sites in the downstream 100 nt intronic flank was not discriminative.

The conservation around the splice site, as measured by the number of human-mouse identical positions in a window of 12 nt (6 on either side) around the exon boundaries, is a highly discriminative feature, despite other features already capturing both conservation information as well as splice site strength. It is interesting that while alternative exons have weaker splice sites, they have stronger conservation around the splice junctions. While only 17.6% of the constitutive exons have identical matches from positions -6 to +6 at the 3'ss, the corresponding figure for alternative exons is as high as 54.7%. At the 5'ss, the corresponding numbers are 30.0% and 60.3%, respectively. This is consistent with a previous study [49].

The GC content of the upstream intronic flank was found to be a useful discriminative feature, and was lower for flanks of alternative exons than of constitutive exons (median values 0.39 vs. 0.42), in agreement with previous studies [25]. However, neither the GC content of the exon nor of the downstream intronic flank was found to be discriminative.

We tested if the di- and trimer (2 and 3 nt words) frequency in exons and intron flanks is different for alternative and constitutive exons. We found that the frequency of di- and trimers in exons is often much more discriminative than the intronic di- and trimer frequencies. This suggests that splice regulatory elements governing exon skipping are more common in alternative exons than in introns flanking them.

Apart from introducing novel features, we also used features derived from known features. These combinations were often more informative than the individual features. For example, the ratios of intron lengths to exon length were more informative than the lengths themselves. The ratio of the length of the downstream intron to the exon length was an especially useful feature, suggesting that exon skipping may occur when the spliceosome finds it difficult to accurately "spot" an exon upstream of a relatively much longer intron.

Splice site strength, first used by [10], was also found be a discriminative feature, with alternative exons having both weaker 3'ss as well as 5'ss than constitutive exons (median scores 7.86 vs. 8.76 and 8 vs. 8.68, respectively).

### Most informative features

Next we asked which were the most informative features using the information gain, a well established measure in machine learning. Information gain is the reduction in the entropy of the class variable, given the feature. While information gain is a good measure of the quality of features, it tends to prefer features with a large number of possible values [38]. A measure which avoid this is the information gain ratio, which divides the information by the information of the feature itself, thus penalizing features with a high inherent information. The top ten features according to the information gain and the information gain ratio criteria are given in Table 2. Two features, exon identity and length of best alignment in the upstream intron flank, appear in both lists.

**Table 2:** Top features according to information gain and information gain ratio (excluding combination features)

**Rank**	**Feature**	**Information Gain**	**Feature**	**Information Gain Ratio**
1	Length of best alignment in the upstream intron flank	0.169	Abundance of GA in exon	0.172
2	Upstream intron flank conservation	0.169	Density of single stranded ESEs in exon	0.151
3	Identity of best alignment in the upstream intron flank	0.142	Exon identity	0.128
4	Downstream intron flank conservation	0.138	Average of positive NI scores in exon	0.118
5	Length of best alignment in the downstream intron flank	0.138	Length of best alignment in the upstream intron flank	0.117
6	Exon identity	0.120	Density of AC in exon	0.115
7	Identity of best alignment in the downstream intron flank	0.088	Average of negative NI scores in exon	0.112
8	Exon length	0.080	Density of CT in exon	0.111
9	Matches in 12-mer near 3'ss	0.066	ESE density in exon	0.104
10	Symmetry	0.042	Length of best alignment in the upstream intron flank	0.103

Table 3 shows the top ten combination features according to the information gain criterion. Seven of these are more informative than any of the features that were combined to obtain these features, while the other three (the ratio of the intron lengths and the exon length, and the sum of the number of the two types of PTB binding sites) are more informative than one of the two features, but less than the other. Not surprisingly, the combinations of conservation related features have a very high information gain (top four).

**Table 3:** Top combination features according to information gain

**Rank**	**Feature**	**Information Gain**
1	Product of identities of exon and both intron flanks	0.208
2	Product of identity of both intron flanks	0.196
3	Product of identities of exon and upstream intron flank	0.181
4	Product of identities of exon and downstream intron flank	0.153
5	Ratio of the downstream intron length to exon length	0.051
6	Ratio of ESE density to ESS density	0.029
7	Sum of splice site scores	0.023
8	Ratio of the upstream intron length to exon length	0.022
9	Ratio of trusted ESE density to trusted ESS density	0.010
10	Density of putative PTB binding sites in exon	0.008

Table 4 shows the ten most informative trimers in the exon and in the intronic flanks according to information gain. Note that the trimers in the exon have a higher information gain, a trend which is also true when looking at all possible 64 trimers in the exon and the intronic flanks. This disagrees with the conclusion of the previous study [8], which used a different feature ranking criterion.

**Table 4:** Top trimers in the exon and intron flanks according to information gain

**Rank**	**Exon Trimer**	**Information Gain**	**Intron**	**Trimer**	**Information Gain**
1	TCC	0.034	upstream	TTC	0.016
2	ATG	0.031	downstream	AGG	0.014
3	CCT	0.029	downstream	GAG	0.012
4	TCG	0.028	upstream	TTT	0.012
5	CAT	0.028	upstream	TCT	0.012
6	AAG	0.027	downstream	GGA	0.012
7	GTA	0.027	downstream	TTT	0.011
8	GAC	0.026	upstream	GAG	0.011
9	GAT	0.026	upstream	AGG	0.011
10	CAA	0.026	upstream	CAG	0.009

### A Bayesian network with an optimized set of 34 features

**Figure 2:**
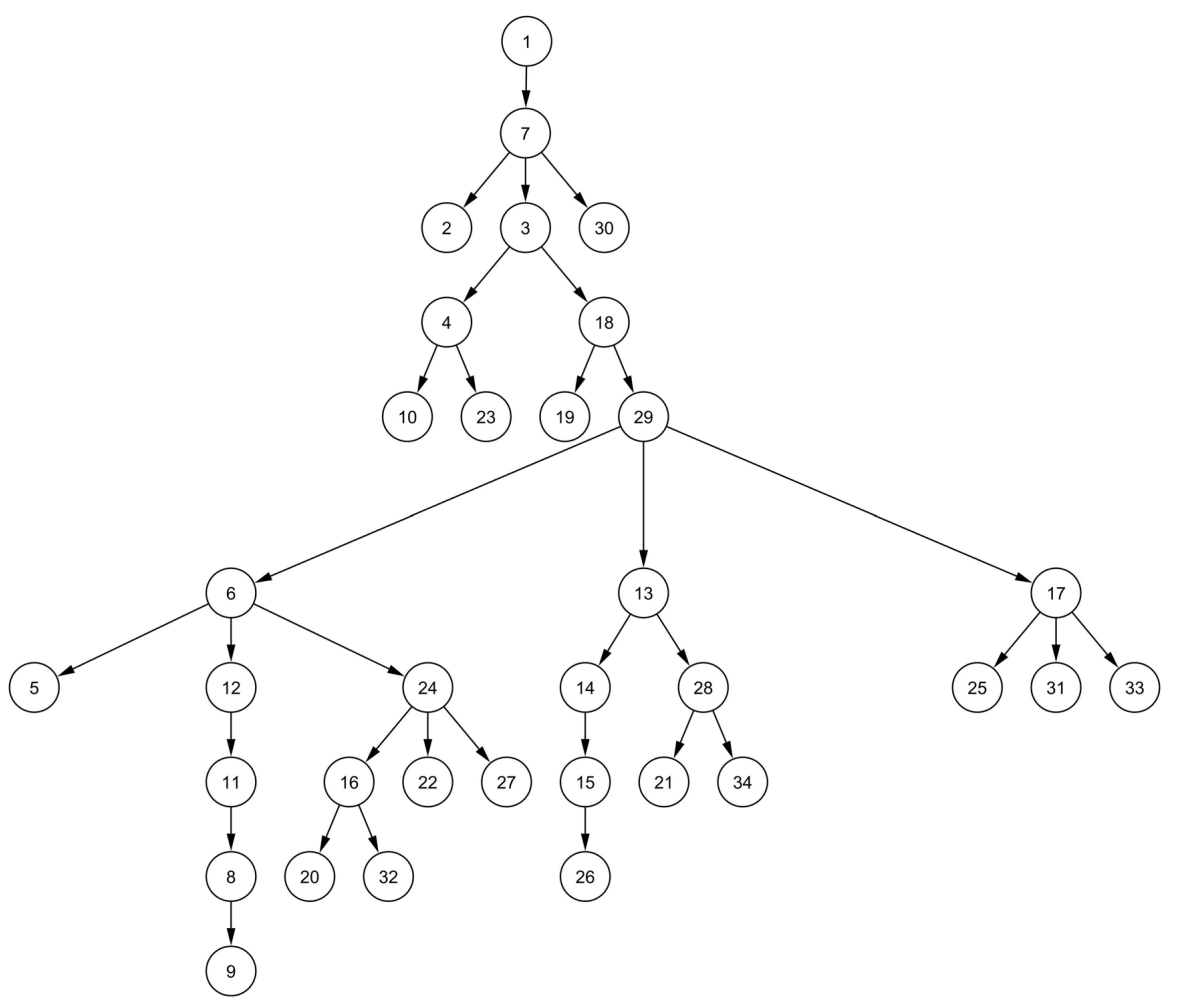
**34-feature Bayesian network.** Note that BN in fact has 35 nodes. The class node, which has an edge to all other nodes and makes the actual number of edges 67, is omitted for ease of visualization. Thus, this is just the augmenting tree in the TAN classifier. The features associated with the nodes are as follows: 1: 1 if exon length is divisible by 3, otherwise 0. 2: Length of the best alignment in the 3' 100 nt intronic region. 3: Length of the best alignment in the 5' 100 nt intronic region.4: Percent identity of the best alignment in the 5' 100 nt intronic region. 5: Length of the 5' intron. 6: Ratio of the lengths of the 3' intron and the exon. 7: Product of the identities of the exon and both 100-nt intronic flanks with their mouse orthologs. 8: 1 if G at +4 of the 5'ss, otherwise 0. 9: T at +4, 10: A at +6; 11: MAXENTSCAN score of the 5'ss. 12: Sum of the MAXENTSCAN scores of the 3' and 5'ss. 13: Average of the NI scores of all the hexamers with a negative NI score. 14: Variance of the NI scores of all the hexamers with a "strong" (≥ 0.8 or ≤ -0.8) score.   15: Average of the NI scores of all the hexamers with a "strong" (≤ -0.8) negative score. 16: Density of single-stranded (PU value ≥ 0.6), "trusted" ESEs (NI score = 1). 17: Ratio of the number of "trusted" ESEs (NI score = 1) to the number of ESSs (NI score = -1). 18: Density of ISREs enriched in the flanks of AS exons, in the 5'intron flank. 19: Density of single-stranded (PU value ≥ 0.6), intronic splice regulatory elements (ISREs) enriched in the flanks of AS exons, in the 5'intron flank. 20: PTB-binding site TCTT density in the exon. Dimer density in the exon:21:CC, 22: GA; 23: Dimer GA density in the 3' intron flank; Trimer density in the exon: 24: AAG, 25: AGG, 26: ATG, 27: CAA, 28:CCA, 29: CGG, 30: CTC, 31: GCA, 32: GGT, 33: TAG, 34: TCC.

All three methods of feature selection available in the BioBayesNet framework were used to arrive at an optimized subset of 34 features. A performance of TP_0.5 _= 61% (65%, 61%, and 56% for the 3-fold cross-validation), and AUC = 0.94 (0.94, 0.94 and 0.94) was achieved using the same subset of 34 features with each fold. The BN learned on the entire dataset with the same features, with 34 nodes and 33 edges, can be seen in Fig. 2. We would like to point out some interesting edges in this network which confirm and may extend our biological knowledge of the splicing process:

- *"length of the best local alignment of the upstream intron flank and its mouse ortholog" (node 3) and "density of intronic splice regulatory elements (ISREs) enriched in introns flanking AS exons, in the upstream intron flank" (node 18)*: Since alternative conserved exons (ACE) tend to have longer conserved regions and a higher density of ISREs in their intron flanks, this is a biologically meaningful dependence.

- *"ratio of the lengths of the downstream intron and the exon" (node 5) and "sum of the MAXENTSCAN scores of the 3' and 5' splice sites" (node 12)*: ACEs tend to have high ratios of intron to exon lengths, and weak splice sites, when compared to constitutive exons [10].

- *"density of single-stranded ESEs" (node 16) and "density of TCTT in exon" (node 20)*: ACEs are enriched in exons with multiple occurrences of TCTT, which is a binding site of the splice-repressor, PTB [36], and tend to have a lower density of single-stranded ESEs when compared to constitutive exons.

- *"MAXENTSCAN score of the 5'ss" (node 11) and nodes representing positions in the 5'ss region (nodes 8 and 9)*: The node representing the strength of the 5'ss has an edge to the node representing the binary variable "Gat5SSplus4", which indicates whether the nucleotide at the position +4 is a G or not, and this node has an edge to the node representing the variable for a T at position +4. Both of these nucleotides are different from the splice site consensus at their respective positions, and thus contribute to lowering the splice site score. Furthermore, it is also known that there are dependencies among the 5'ss positions [50].

- *"density of intronic splice regulatory elements (ISREs) enriched in intronic flanks of AS exons, in the upstream intron flank" (node 18) and "Abundance of CGG in the exon" (node 29)*: ISREs which are enriched in the flanks of alternative exons tend to be CG-rich [30], so the link to CGG motifs in the exon might indicate a subclass of alternative exons found in CG-rich regions.

- *"abundance of CCA in the exon" (node 28) and "average of negative NI scores in exon" (node 13)*: These nodes correspond to features representing the density of the trimer CCA in the exon, and the average NI score of all hexamers with negative NI scores, i.e. ESS-like tendencies. The CCA motif is ~35-fold less frequent in ESSs than ESEs (occurs in 71 of 979 ESEs and only 1 of 496 ESSs), so the BN captures the association of CCA abundance with the average of negative NI scores.

- *"abundance of CGG and GCA in the exon" (nodes 29 and 31) and "ratio of ESEs to ESSs in the exon" (node 18)*: These nodes represent the density of the trimers CGG and GCA in the exon, and the ratio of "trusted" ESEs and ESSs (scores of 1 and -1, respectively). The motif CGG occurs in 7.5% (50 of 666) of the trusted ESEs, but in only 1% (4 of 386) of the trusted ESSs. Similarly, the motif CGG occurs in 10.5% (70 of 666) of the trusted ESEs, but in only 2.1% (8 of 386) of the trusted ESSs. Thus there is a correlation between the abundance of the motifs CGG and GCA in the exon, and the ratio of ESEs to ESSs.

Some of the other edges can be explained in a trivial manner, for instance those involving the density of overlapping motifs (e.g. nodes 28 and 21, and 28 and 34). We note that one must be careful in interpreting the edges, as not all of them may lend themselves to meaningful biological interpretation. While not all edges can be interpreted with biological knowledge, they definitely help in our classification since a classifier omitting all edges (naïve Bayes) performs worse [8].

### Comparison of 2-fold, 3-fold, 5-fold, and 10-fold cross-validation

We used 3-fold cross-validation in order to compare our results to [8], who did the same. However, since it is common in machine learning to use 2-fold, 5-fold, 10-fold, or "leave one out" (LOO) cross-validation, we compared the performance of these different approaches on the dataset D1, using the WEKA package [38], and the optimized set of 34 features described above. The results for the 2/3/5/10/LOO cross-validations were: TP_0.5 _= 57%/60%/57%/58%/59%, and AUC = 0.95/0.95/0.95/0.95/0.95.

### Performance using the same features as the SVM

To assess the factors behind the improved performance of BNs, we used the same 228 features as reported in [8], and obtained the same overall quality of prediction (AUC = 0.93) and slightly improved TP_0.5 _(51% vs. 50%). This indicates that accurate classification of conserved exon skipping depends more on the features used rather than the choice of classifier.

### Performance of Bayesian networks on a second dataset

Next, we tested our approach on a different dataset of conserved exon skipping events, the ACESCAN training set [10] henceforth called dataset D2, which comprises 5,069 constitutive and 241 alternative exons. Using the basic set of 228 features [8], the BN achieved values of TP_0.5 _= 52%, and AUC = 0.92. After incorporating the novel features, and performing feature selection as described above, the best performance achieved on D2 was: TP_0.5 _= 59%, and AUC = 0.93 (Figure 1).

Thus, we achieve a good performance, similar to that on D1, on the dataset D2 as well. However, the number of discriminative features is smaller than for D1. This trend continues with the addition of novel features – of all the 365 features, typically 110–130 are discriminative on a 2/3 split of D1, whereas only 65–80 are discriminative on a 2/3 split of D2. A possible reason for this could be the different criteria used in the construction of the two datasets, resulting in possibly different extents of corruption of the sets of constitutive exons by alternative exons, because the dataset D1 requires 4 identical ESTs for an exon to be considered constitutive, whereas the dataset D2 does not. Furthermore, D1 has more exaggerated differences among the two classes for several features – for example, while 74% of alternative exons preserve the reading frame compared to 37% of the constitutive exons, the corresponding numbers for D2 are 67% and 39%. Thus, the subset of conserved exon skipping events in D1 seems to be characterized by more strongly discriminative features.

### Cross-validation by training and testing on the two independently constructed datasets

It is usual in machine learning to divide the available data into training and testing partitions, and optimize the classification using these. It is then assumed that similar performance can be achieved on other datasets of a similar nature. However, given that there are often differences in the way independent datasets are prepared by different groups of scientists, it may be optimistic to presume this. We suggest that testing on an independent dataset is likely to give a better indication of the level of performance that can be expected when scaling to a genome-wide prediction. To use D1 and D2 for this purpose, we removed from D1 the exons already present in D2 – leaving 201 alternative and 1,654 constitutive exons in D1. To minimize any biases introduced by different ratios of the numbers of samples in each class, we then randomly sampled constitutive exons from D2 to have the same ratio (8.23:1) of constitutive to alternative exons, leaving 241 alternative and 1,984 constitutive exons in D2. We then used the optimal feature subsets obtained on D1 and D2 earlier to train BNs on the respective entire datasets. When we used the BN trained on D1 to test D2, the performance achieved was TP_0.5 _= 27%, and AUC = 0.88. The corresponding performance achieved with training on D2 and testing on D1 was TP_0.5 _= 26%, and AUC = 0.91. While an AUC value of 0.91 (or even 0.88) indicates good overall classification, this is less than the 0.94 achieved when tested on unseen data from the same source. The effect on TP_0.5 _is quite dramatic. We think that these figures might be a more accurate estimate of what to expect when a classifier is used to classify independently produced data. Performance will tend to be (at least) slightly worse on independently produced data than on unseen data from the same source, something which is true of all classifiers in general.

### Assessing over-fitting

To assess whether our increased performance is due to over-fitting, we randomly permutated the labels 'alternative' and 'constitutive' between the data points and trained the BN on the relabelled datasets D1 and D2. In case of overfitting, we would still expect a good performance, while the AUC value of a random classifier should be close to 0.5 [38].

After relabelling, most features are no longer discriminative. In fact, only 29 features remained discriminative, and these were the same for both datasets – symmetry, and the 28 features describing the positional biases in the 5'ss region. The AUC achieved was 0.51 on dataset D1, and 0.49 on D2. This shows that our approach has no problems with over-fitting.

To further rule out overfitting, we used a random three way split: 60% of the data for training, 20% for validation and optimization, and 20% for testing. We obtained TP_0.5 _= 63% and AUC = 0.94 on the validation set; using the same set of features, the performance on the test set was TP_0.5 _= 59% and AUC = 0.95. Using this "train-validate-test" approach on D2, we obtained TP_0.5 _= 58% and AUC = 0.94 on the validation set, and TP_0.5 _= 60% and AUC = 0.93 on the test set. Since the performance on both datasets is very similar to the performance achieved using our three-stage feature selection approach, we conclude that the improvement is not mainly due to manual feature selection. However, manual selection is not ideal, and an automated feature selection algorithm designed to optimize performance in the low false-positive region would be more satisfying. This is one of the possible future directions of work.

As a first approach to entirely automated feature selection, we performed the following experiment: we randomly chose 75% of D1 for training, and 25% for testing. Feature selection was done using only the training part, and the test part was touched only once at the very end of the procedure. The feature selection was as follows: starting with the full set of features, we iteratively discarded one feature at a time, and performed 10-fold cross-validated classification using a BN (TAN) with the remaining features. Features were discarded in order of increasing information gain, that is, the least informative features were discarded first. We re-inserted a feature only if at least one of TP_0.5 _or AUC decreased as a result of omitting it. This was done only in one pass, and features once discarded, were not considered again. This is clearly not an optimal strategy, and leads to bigger feature subsets than the approach used before, but still yields good results. Using the subset of 50 features thus obtained on D1 led to performance of TP_0.5 _= 54% and AUC = 0.94 on the training set, and TP_0.5 _= 57% and AUC = 0.91 on the test set. On D2, this approach yielded a subset of 35 features and a performance of TP_0.5 _= 56% and AUC = 0.92 on the training set, and TP_0.5 _= 52% and AUC = 0.94 on the test set. Thus, we can also obtain good performance on unseen data using a feature selection strategy which, though suboptimal, is easy to automate.

Moreover, we also used the feature sets obtained in the "train-validate-test" setting with a naïve Bayes classifier (NBC) and obtained TP_0.5 _= 47% and AUC = 0.93 for a 10-fold cross validation on D1, and TP_0.5 _= 43% and AUC = 0.92 for a 10-fold cross validation on D2, which are both better than the performance using NBC reported in [8] (TP_0.5 _= 37% and AUC = 0.89). Compared to the BNs, NBCs achieve a higher sensitivity but lower specificity. This indicates that the novel features help in improving classification performance, and similar improvements should be possible using other classifiers like SVMs, Neural networks and so on.

### False positives with high posterior probability are likely true alternative exons

Next, we carefully looked at exons that are labelled constitutive but obtained a high posterior probability of being alternative exons from the BN. Since they seemed to be more similar to ACEs than to other constitutive exons, we hypothesized that newer EST/cDNA data provides evidence for exon skipping, or any other kind of alternative splicing at these exons. Out of 1,753 exons in D1 that were labelled constitutive, 14 were assigned a P(ACE) – posterior probability of being an ACE – of 0.7 or more. A detailed inspection using the UCSC genome browser [22] revealed that seven have EST and/or mRNA evidence of alternative splicing in at least one of human and mouse (six of these seven are cassette exons) and that two of them are alternatively skipped in both species, that is, have evidence of being ACEs. Of the remaining seven exons, one has evidence of being a cassette exon in orangutan (Additional file 1).

The results on D2 are even more impressive – there are 15 exons labelled constitutive and with P(ACE) ≥ 0.7, of which 13 have evidence of exon skipping or another alternative splicing event (seven are cassette exons in at least one of human and mouse; five are ACEs; Additional file 1).

Thus, most FP predictions with high posterior probabilities of being cassette exons in both D1 and D2 datasets are actually alternative despite being labelled constitutive at the time the datasets were prepared. This further demonstrates the good performance of the BN.

### Predicting exon skipping without using conservation based features

While conservation based features have proved to be the most discriminative, it is desirable to be able to predict alternative splice events using only features that are available to the spliceosome. The performance on this test is also indicative of our understanding of the process of exon skipping. Hence, we should also aim to predict splicing using only information available from a single genome. We predicted exon skipping omitting all conservation based features – the best performance achieved was TP_0.5 _= 29%, and AUC = 0.86 on dataset D1 and TP_0.5 _= 26%, and AUC = 0.88 on dataset D2.

While this performance is noticeably poorer than that achieved using conservation based features, we would like to note that the datasets D1 and D2 consist of exons that are either constitutively spliced in both human and mouse, or cassette exons in both. Thus, we are still distinguishing only between conserved constitutive splicing and conserved exon skipping, leaving out cases of species-specific splicing, as well as of alternative splicing of species-specific exons, which form the majority of alternative exons [51].

## Conclusion

Using Bayesian networks (BNs) and several novel features that emerged from recent studies of alternative splicing, we have achieved considerably improved classification of conserved cassette exons. We were able to improve the performance described in [8] due to the incorporation of novel features. To the best of our knowledge, this is the first time that features involving secondary structure and intronic splice regulatory elements have been employed for distinguishing alternative exons from constitutive ones. We also compared our performance on two datasets, and showed that the BN is able to produce accurate classification on both. However, it is worth noting that these datasets differ with respect to discriminative properties.

One direction of future work would be to consolidate various datasets of constitutive and alternative exons, and compile sets of features, which are discriminative over each of them, and the intersection of these sets, which is discriminative over all datasets. Another interesting line to pursue is to predict other kinds of alternative splicing. Here, we focused on exon skipping, which is the most prevalent form of alternative splicing in human and higher vertebrates. However, other major forms of alternative splicing such as alternative donor and acceptor sites [52-54] are also of biological importance, and it would be worthwhile to develop similarly accurate classifiers for these events.

Ideally, we should be able to predict splicing outcomes without conservation based information, as the information required by the spliceosome is present in the given genome. We report our performance at this task, while it is a promising beginning, clearly there is much work to be done. It should be noted that we have ignored two prominent subclasses of alternative exons – namely orthologous exons which are alternatively spliced in a species-specific manner, and species-specific exons which are alternatively spliced. Both these classes are potentially quite important: as up to 50% of all human alternative exons may be human-specific, and up to 60% of all conserved exons which are alternatively spliced may be alternative in a species-specific manner [51]. Classifiers for these tasks are yet to be developed.

## Authors' contributions

RS designed new features, performed feature extraction, used BioBayesNet to perform the classification, did the analyses using WEKA, analyzed results, and drafted the manuscript. MH helped with feature design, extraction of secondary structure related features, analysis of the results, and manuscript development. RP implemented the BioBayesNet webserver, which was the framework for using BNs, and helped by providing various options for feature discretization, feature selection, learning BNs and evaluating performance. UG and MP participated in the analysis of the results, and helped draft the manuscript. RB participated in feature design and helped draft the manuscript. RB and MP supervised the study. All authors have read and approved the final manuscript.

## Supplementary Material

Additional file 1 **The top false positives in the datasets D1 and D2.** Results of investigating the top false positives (P(ACE) = 0.7) in the datasets D1 and D2, using the UCSC genome browser (human genome build hg18, mouse genome build mm9). Click here for file
